# Clinical, microbiological, and immunological aspects of healthy versus peri-implantitis tissue in full arch reconstruction patients: a prospective cross-sectional study

**DOI:** 10.1186/s12903-015-0031-9

**Published:** 2015-04-01

**Authors:** Javier Ata-Ali, Antonio Juan Flichy-Fernández, Teresa Alegre-Domingo, Fadi Ata-Ali, Jose Palacio, Miguel Peñarrocha-Diago

**Affiliations:** Public Dental Health Service, Arnau de Vilanova Hospital, San Clemente Street 12, 46015 Valencia, Spain; Oral Surgery and Implantology, Valencia University Medical and Dental School, Valencia, Spain; Valencia University Medical and Dental School, Valencia, Spain; Immunology Unit, Institute of Biotechnology and Biomedicine, University of Barcelona, Barcelona, Spain

**Keywords:** Peri-implantitis, Cytokine, Review, Periodontal pathogens, Peri-implant diseases, Dental implant, Peri-implant sulcus fluid (PISF)

## Abstract

**Background:**

Due to the world-wide increase in treatments involving implant placement, the incidence of peri-implant disease is increasing. Late implant failure is the result of the inability to maintain osseointegration, whose most important cause is peri-implantitis. The aim of this study was to analyze the clinical, microbiological, and immunological aspects in the peri-implant sulcus fluid (PISF) of patients with healthy dental implants and patients with peri-implantitis.

**Methods:**

PISF samples were obtained from 24 peri-implantitis sites and 54 healthy peri-implant sites in this prospective cross-sectional study. The clinical parameters recorded were: modified gingival index (mGI), modified plaque index (mPI) and probing pocket depth (PPD). The periodontopathogenic bacteria *Tannerella forsythia*, *Treponema denticola* and *Porphyromonas gingivalis* were evaluated, together with the total bacterial load (TBL). PISF samples were analyzed for the quantification of Interleukin (IL)-8, IL-1β, IL-6, IL-10 and Tumor Necrosis Factor (TNF)-α using flow cytometry (FACS).

**Results:**

The mGI and PPD scores in the peri-implantitis group were significantly higher than the healthy group (p < 0.001). A total of 61.5% of the patients with peri-implantitis had both arches rehabilitated, compared with 22.7% of patients with healthy peri-implant tissues; there was no implant with peri-implantitis in cases that received mandibular treatment exclusively (p < 0.05). Concentrations of *Porphyromonas gingivalis* (p < 0.01), association with bacteria *Porphyromonas gingivalis* and *Treponema denticola* (p < 0.05), as well as the TBL (p < 0.05) are significantly higher in the peri-implantitis group. IL-1β (p < 0.01), IL-6 (p < 0.01), IL-10 (p < 0.05) and TNF-α (p < 0.01) are significantly higher at the sites with peri-implantitis compared to healthy peri-implant tissue, while IL-8 did not increase significantly.

**Conclusion:**

The results of the present study involving a limited patient sample suggest that the peri-implant microbiota and which dental arch was rehabilitated involved could contribute to bone loss in peri-implantitis. A significant relationship is observed between the concentration of cytokines (interleukins 1β, 6 and 10 and TNF-α) and the inflammatory response in peri-implantitis tissue.

## Background

The restoration of missing teeth by means of dental implants has now become a routine treatment in common use [1]. Various longitudinal studies have shown the high survival rates of implants in functional use, which range between 90% and 95% over a follow-up period of up to 20 years [2-4]. Peri-implant disease infection etiology has been described in detail in the literature for both mucositis [5-8] and peri-implantitis [9-12]. Late implant failure is the result of the inability to maintain osseointegration, whose most important cause is peri-implantitis [1].

Using the checkerboard DNA-DNA hybridization method, Socransky et al. [13] identified a consortium of the bacterial species *Tannerella forsythia (T. forsythia)*, *Treponema denticola (T. denticola)* and *Porphyromonas gingivalis (P. gingivalis)* as having the highest association with periodontal disease severity. The authors named this microbial consortium “red complex”. Evaluation of the literature has shown the microbiota associated to peri-implantitis to be more complex than that found under healthy peri-implant conditions – the main flora consisting of anaerobic gram-negative bacteria [14]. A high degree of association between this red complex and the appearance of peri-implantitis has been observed [9-12,14]. However, in healthy peri-implant sulci, oral streptococci constitute the predominant bacterial flora [15].

Although different cytokines have been evaluated [16-18], the cytokine concentrations that differentiate between healthy and stable sites and the onset of a pathological periodontal and peri-implant process are not known [19]. In the periodontium, individual differences in inflammatory and immunological responses to bacterial infection may influence the host’s susceptibility to disease [20]. The cascade of inflammatory mediators of the host in response to bacterial infection, which can result in destruction of connective tissue and bone, is determined by genetic factors [21].

The aim of this study was to study the clinical characteristics of peri-implantitis as established by the modified plaque index, modified gingival index and probe depth, examines the microbial and host response Interleukin (IL)-8, IL-1β, IL-6, IL-10 and Tumor Necrosis Factor (TNF)-α characteristics in dental implants with peri-implantitis, and establishes comparisons with healthy dental implants.

## Methods

### Study population

**Figure 1:**
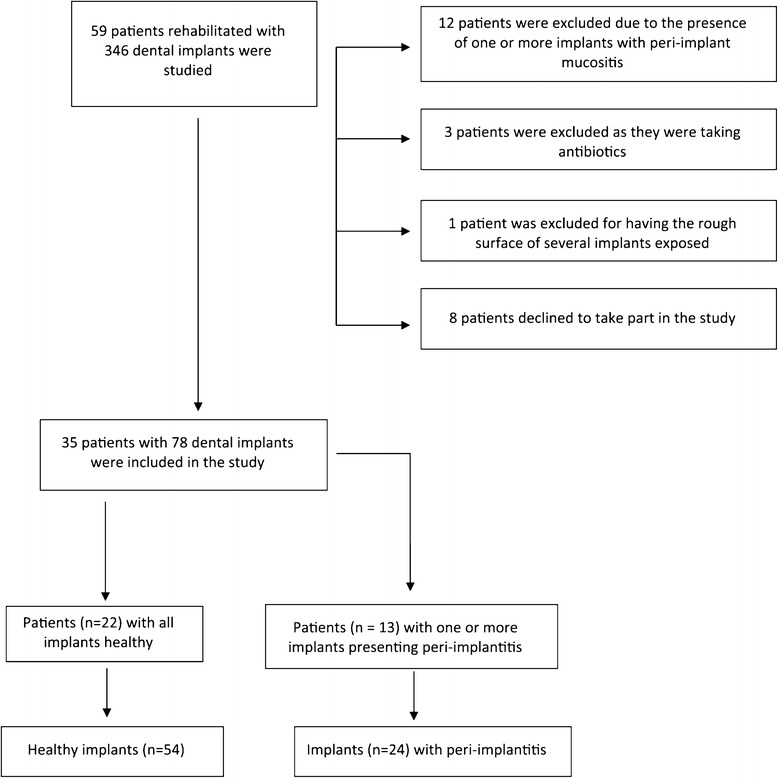
**Diagram showing patients included and excluded from the study.**

This is a prospective cross-sectional study of clinical, microbiological and immunological markers of 24 peri-implantitis and 54 healthy peri-implant sites. Sixty-six patients were treated at the Department of Oral Surgery and Implantology at Valencia University Medical and Dental School (Valencia, Spain). All patients presented at least one completely edentulous dental arch, which were rehabilitated with dental implants (Figure 1). All patients were seen by a single examiner (JAA). The study was conducted in accordance with the Helsinki Declaration and the protocol was approved by the institutional review board of the University of Valencia; patients gave their informed consent to participate in the study in writing.

We excluded those patients who had received any kind of local or systemic decontamination treatment of the oral cavity in the previous three months (such as antibiotics or rinses), or periodontal treatment in the previous six months. Patients with uncontrolled periodontal disease (assessed by a specialist in periodontics) were also excluded, in the same way as individuals presenting implants with exposure of the rough surface, patients with systemic diseases (e.g., HIV infection or leukemia) or who were in receipt of drugs capable of altering gingival health in some way, or pregnant women and nursing mothers (Figure 1).

The study population consisted of 35 individuals (22 patients with healthy implants and 13 with peri-implantitis). Seventy-eight dental implants were evaluated during the study (24 with peri-implantitis and 54 healthy peri-implant sites). Phibo® treatment surface Avantblast (TSA) implants (Phibo Dental Solutions, Sentmenat, Barcelona, Spain) had been implanted in the patient sample, and all implants had been in functional use for at least 24 months. The data collected was analyzed, relating them to age, sex, smoking, oral hygiene, which arch had been rehabilitated and the type of prosthesis (Table 1).

**Table 1:** **Demographic and clinical description of the study population**

	**Healthy**	**Peri-implantitis**	**Differences per group**
Age (mean ± s.d.)	63.6 ± 10.4	52 ± 7.7	**
Gender (% females) Number of patients Number of implants	59.1 22 54	53.8 13 24	n.s.
Smoking habit Non-smokers (%)	100.0	38.5	**
Smokers (%)	0.0	61.5	
Oral hygiene Never (%)	0	7.7	n.s.
1-2 times/day (%)	63.6	46.2	
3 times/day (%)	36.4	46.2	
Rehabilitated arch Upper (%)	31.8	38.5	*
Lower (%)	45.5	0	
Both (%)	22.7	61.5	
Prosthesis^1^ Fixed (%)	31.8	38.5	n.s.
OD Locator® (%)	45.5	7.7	*
OD Bar (%)	9.1	38.5	*
Fixed and OD Locator (%)	4.5	0	
OD Locator and OD Bar (%)	4.5	7.7	
OD Bar and hybrid (%)	4.5	0.0	
Hybrid (%)	0.0	7.7	

Chi-squared test for evaluating differences in gender and type of prosthesis between groups.

Mann-Whitney *U*-test for evaluating differences in smoking, brushing and arch between groups.

Student *t*-test for evaluating age differences between groups.

Note: Only differences in the proportion of the three most frequent types of prostheses are evaluated.

n.s. = nonsignificant.

OD = overdenture; Locator® (Zest Anchors, Escondido, CA, USA).

s.d. = standard deviation.

*p < 0.05; **p < 0.01.

### Implant inclusion criteria

The patients were divided into two groups according to whether or not they presented peri-implantitis. Peri-implantitis was defined according to Schwarz et al. [22]: implant with a probing depth ≥4 mm and signs of acute peri-implantitis (loss of supporting bone as estimated on radiographs, bleeding on probing or suppuration) and no implant mobility. The inclusion criteria in the group of patients with healthy dental implants were: probing pocket depth (PPD) < 4 mm [17,23,24], absence of clinical signs of inflammation of the peri-implant mucosa, and without radiographic bone loss. If one of the implants was healthy but another showed signs of peri-implantitis, the patient was classified as having the disease.

The implant with the deepest peri-implant pocket was selected for collection of the microbiological samples and for determining interleukins, selecting one implant from each rehabilitated quadrant. When there were two or more implants with the same probe depth, the most anteriorly positioned implant was selected.

### Clinical examination

We examined all the implants in each patient, recording the implant data per rehabilitated quadrant in each subject (registering two or four implants according to whether the upper maxilla, the mandible, or both were rehabilitated). The modified gingival index (mGI) and the modified plaque index (mPI) were determined for each implant according to methods described by Mombelli et al. [25]. The peri-implant probing pocket depth (PPD) was measured with a probe calibrated to 0.25 Nw (Click Probe, Kerr, USA). Specifically, PPD was measured at the mesiobuccal, mediobuccal, distobuccal, mesiolingual, mediolingual and distolingual points of each implant, and the mean PPD was calculated for each implant [26].

### Radiographic evaluation

Marginal bone loss was measured from the intraoral X-ray studies, using the XMind® intraoral system (Groupe Satelec-Pierre Rolland, Bordeaux, France) and the RVG® intraoral digital receptor (Kodak Dental System, Atlanta, GA, USA). The XCP® X-ray positioning device (Dentsply, Des Plaines, IL, USA) was used to reproduce the angle of the X-rays in later reviews. In order to position the XCP® correctly, the guide bar was placed parallel to the direction of the X-ray beam, perpendicular to the digital receptor. According to the VII European Workshop in Periodontology, to establish the baseline, a radiograph should be obtained to determine alveolar bone levels after physiological remodeling, and peri-implant probing assessments should not be performed before this has taken place as it is assumed that bone loss occurring after initial remodeling is mainly due to bacterial infection [27].

### PISF sampling

Peri-implant sulcus fluid (PISF) was collected from each implant after the presence or absence of plaque (mPI) had been assessed and before registering any other clinical parameters [26]. After calibrating the Periotron® 8000 (Proflow Incorporated. New York, USA), the PISF sample was collected with sterile paper strips (Periopaper Strip® Proflow Incorporated. New York, USA).

The technique used was as follows: a) drying the mouth with aspiration; b) isolation of the zone with cotton rolls; c) gentle drying of the zone; d) sulcus fluid sampling by placing the sterile paper strips in the sulcus between the implant and gums, keeping this position for 30 seconds; e) placement between the sensors of the Periotron® 8000, to record the amount of PISF obtained in Periotron units previously calibrated following the manufacturer’s indications.

PISF was absorbed by each strip. Each sample was diluted in an Eppendorf tube with 200 μL of 50 mM phosphate buffer, pH 7.2, together with a pool of protease inhibitors (Roche Diagnostics GmbH, Mannheim, Germany) and 0.1 mM phenyl sulfonyl fluorate, and incubated for two hours. The samples were centrifuged at 1000 g for five minutes, and the supernatant was stored at -80°C until used.

### Cytokine assay

IL-1β, 6, 8, 10 and TNF-α cytokines were evaluated in the supernatants stored at -80°C. The evaluation was performed by using the Human Inflammation Cytometric Bead Array (CBA) system (Becton Dickinson, BD Biosciences, San Diego, CA, USA) and FACS analysis (Becton Dickinson, BD Biosciences, San Diego, CA, USA). The samples and positive controls (standard curve) were processed according to the manufacturer’s instructions, and the values for cytokines were calculated and reported as pg/ml. Data were acquired with a FACS calibur flow cytometer (Becton Dickinson, Franklin Lakes, NJ, USA).

### Microbiological sampling

Supragingival plaque was removed from the implant with the deepest peri-implant pocket in each quadrant using a curette or cotton roll, without penetrating the gingival sulcus. Cotton rolls were used for relative isolation. The sampling site was dried with an air pistol. Sterile paper tips (Johnson & Johnson, Medical Inc., Arlington, TX, USA) were inserted in the peri-implant sulcus for 10 seconds. The paper tips were then thoroughly impregnated in a solution of guanidine thiocyanate 4 M and 2-mercaptoethanol contained in a tube. For microbiological analysis, the samples were sent to IAI, Inc., where analyses were made of *T. forsythia*, *P. gingivalis*, *T. denticola* and the total bacterial load (TBL) using the IAI-PadoTest 4.5® (IAI Inc., IAI Institute, Zuchwill, Switzerland), a method used by other researchers [28-30]. To this effect, the samples were mounted in nylon membranes and hybridized with specific P32 probes directed against the sRNA ribosomal subunit of the three above mentioned periodontal bacterial species.

### Statistical analysis

The SPSS version 15.0 statistical package for Microsoft Windows (SPSS Inc., Chicago, IL, USA) was used for statistical analysis. Tables 1 and 2 show the statistical tests used for each measure. Statistical significance was considered for p < 0.05. The statistical power reached by the Mann-Whitney *U*-test (used in comparing the 24 implants with peri-implantitis versus the 54 without peri-implantitis) in the bacterial load analysis in the sample of 78 implants was 0.88. A detected effect size of 0.8 was assumed, with a 95% confidence level (α = 0.05).

**Table 2:** **Clinical characteristics of the implants with healthy peri-implant gingiva and with peri-implantitis**

	**Healthy**	**Peri-implantitis**	**Differences per group**
Mean PPD in mm	2.72 ± 0.59	5.15 ± 0.68	*
mPI	0.96 ± 1.03	1.25 ± 1.15	n.s
0 (%)	46.3	37.7	
1 (%)	18.5	16.7	
2 (%)	27.8	29.2	
3 (%)	7.4	16.7	
mGI	0.63 ± 0.92	2.71 ± 0.46	*
0 (%)	63.0	0	
1 (%)	14.8	0	
2 (%)	18.5	29.2	
3 (%)	3.7	70.8	
PISF	91.7 ± 50.3	83.9 ± 43.1	n.s.

Mean ± s.d. or %, as indicated.

*p < 0.001.

Mann-Whitney *U*-test for evaluating differences in PPD, mPI and mGI between groups.

Student *t*-test for evaluating differences in PISF between groups.

n.s. = nonsignificant.

PPD = probe pocket depth; mPI = modified plaque index; mGI = modified gingival index; PISF = peri-implant sulcus fluid.

## Results

Table 1 reports mean patient age (p<0.01), gender, smoking habits (p<0.01), oral hygiene, which arch was rehabilitated (p < 0.05) and prosthetic type (p<0.05) for the two study groups. When prosthetic type was registered, Locator® supporting an overdenture was the most frequent in the patient group with healthy peri-implant sites, while overdentures on bars were the most frequent in implants with peri-implantitis. A total of 61.5% of the patients with peri-implantitis had both arches rehabilitated, compared with 22.7% of patients with healthy peri-implant tissues; there was no implant with peri-implantitis in cases that received mandibular treatment exclusively.

**Figure 2:**
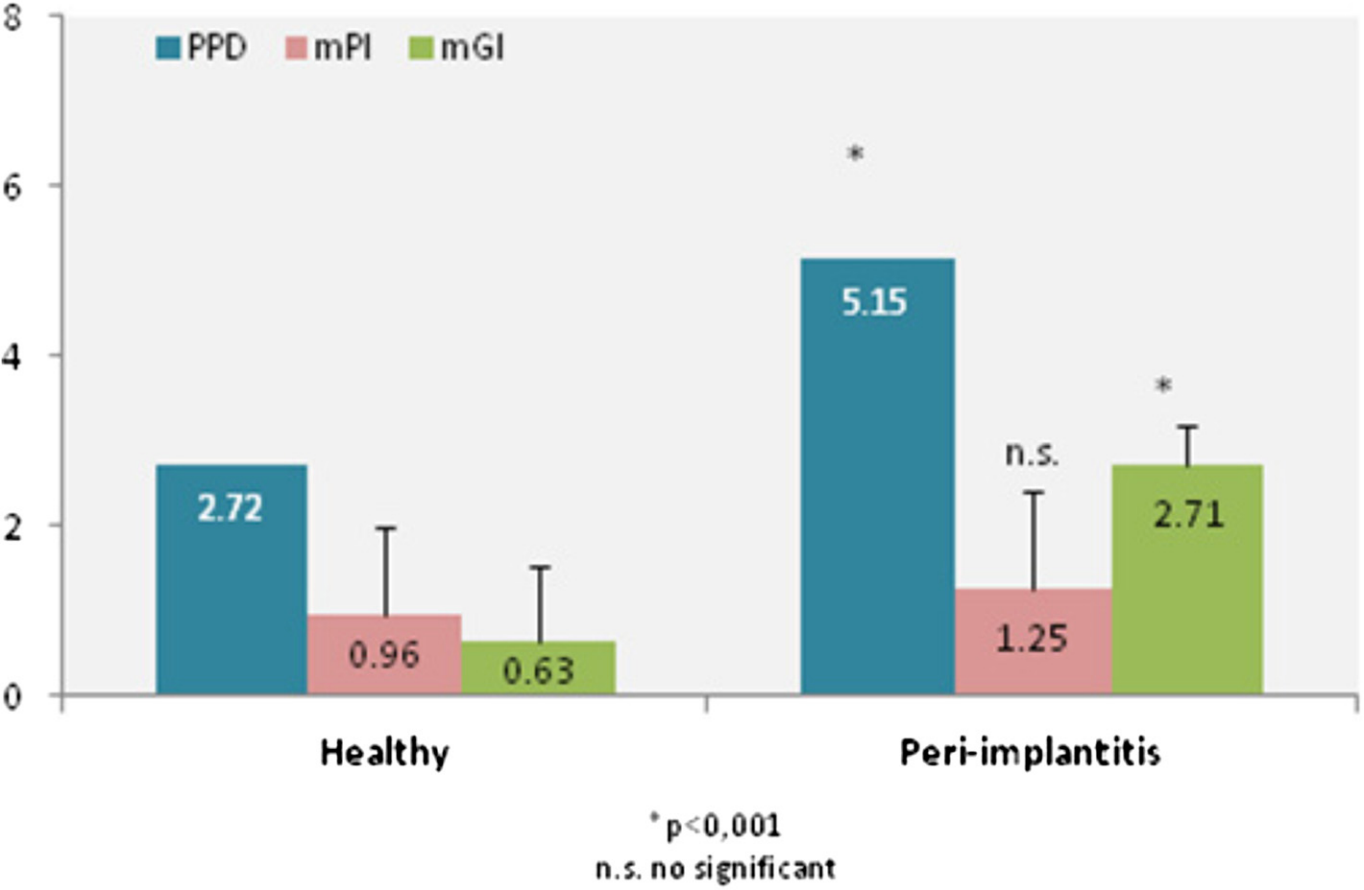
**The mGI and PPD values for the peri-implantitis group were significantly higher than in the healthy group.**

The mean values of clinical parameters for all implants (with or without peri-implantitis) are presented in Table 2. There were no statistically significant differences in the percentage of sites at which plaque was found between the groups. mGI scores were significantly higher around implants with peri-implantitis than around healthy implants (p < 0.001). The recorded mean PPDs in the peri-implantitis group and the healthy group were 5.15 ± 0.68 and 2.72 ± 0.59, respectively, this being a statistically significant difference (p < 0.001) (Figure 2). On examining the PISF volumes, no significant differences were observed between the two groups.

The analysis of putative periodontal pathogens of the red complex (*T. forsythia*, *P. gingivalis*, *T. denticola*) and total bacterial load (TBL) are summarized in Table 3. Subgingival microbiota was composed of a greater number of periodontal pathogens in patients with peri-implantitis, showing significant difference in counts of *P. gingivalis* (p < 0.01), in *P. gingivalis* and *T. denticola* association (p < 0.05), as well as TBL (p < 0.05).

**Table 3:** **Detection frequencies of target bacteria in subgingival peri-implant sites for each group**

	**T. forsythia (Tf)**	**P. gingivalis (Pg)**	**T. denticola (Td)**	**TBL**	**Tf + Pg**	**Tf + Td**	**Pg + Td**	**Red complex**
Healthy	12 (22.2%)	6 (11.1%)	9 (16.7%)	52 (96.3%)	6 (11.1%)	6 (11.1%)	6 (11.1%)	6 (11.1%)
Peri-implantitis	8 (33.3%)	9 (37.5%)	8 (33.3%)	24 (100%)	6 (25%)	6 (25%)	8 (33%)	6 (25%)
Differences per group	n.s.	**	n.s.	n.s.	n.s.	n.s.	*	n.s.

Chi-squared test for evaluating differences in bacterial presence between groups.

n.s. = nonsignificant.

*p < 0.05; **p < 0.01.

Tf: Tannerella forsythia (T. forsythia); Pg: Porphyromonas gingivalis (P. gingivalis); Td: Treponema denticola (T. denticola).

Red Complex = Tf + Pg + Td.

TBL = Total Bacterial Load.

**Figure 3:**
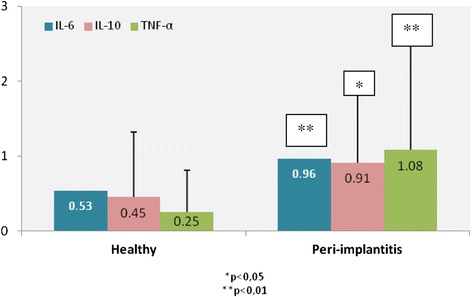
**Differences in IL-6 (p < 0.01), IL-10 (p < 0.05) and TNF-α (p < 0.01) in patients with peri-implantitis and in patients with healthy peri-implant tissues (pg/ml).**

**Figure 4:**
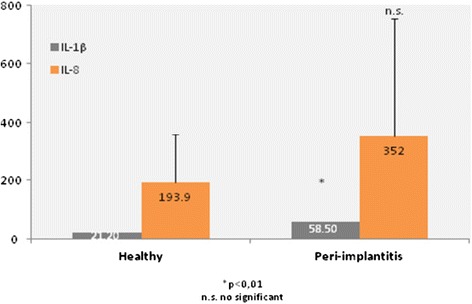
**Differences in IL-1β (p < 0.01) and IL-8 in patients between peri-implantitis and patients with healthy implants (pg/ml).**

The peri-implantitis group showed a significantly greater level of IL-6 than the healthy group (0.96 ± 0.64 and 0.53 ± 0.63 respectively, p < 0.01); IL-1β (58.5 ± 84.8 and 21.2 ± 24.2 respectively, p < 0.01); IL-10 (0.91 ± 0.90 and 0.45 ± 0.87 respectively, p < 0.05); TNF- α (1.08 ± 1.49 and 0.25 ± 0.56 respectively, p < 0.01) (Figures 3 and 4). Although IL-8 increased in the peri-implantitis group, there was no statistically significant difference in comparison with the healthy implant group. The IL-1β/ IL-10 ratio was found to be 9.9 ± 11.9 for the healthy group and 37.2 ± 44.4 for the peri-implantitis group (p = 0.006).

## Discussion

Due to the world-wide increase in treatments involving implant placement, the incidence of peri-implant disease is increasing. Initial screening of peri-implant tissues will consist of an evaluation of peri-implant pocket probing depth and the degree of bleeding on probing [31]. When increased bacterial plaque and bleeding in response to probing affects over 30% of the dental implants, this situation is related to a high risk of mucositis and peri-implantitis [32]. A study [33] involving 34 patients with 77 dental implants (comprising 23 mucositis and 54 healthy periimplant sites) concluded that bacterial plaque induces an inflammatory response that can lead to the development of peri-implant mucositis. A recent systematic review [34] highlights the lack of uniform treatment and the need to establish additional research to fully provide effective treatments for this common condition, which is the first step to prevention peri-implantitis. These data are consistent with those published by Shibli et al. [11], which found patients with peri-implantitis to have increments in all the evaluated clinical parameters, except the percentage of locations with bacterial plaque. mGI score and PPD are parameters that must be evaluated for the diagnosis of peri-implant disease [27,28,32]. Accordingly, the present study found that both mGI and PPD were significantly higher in the implants with peri-implantitis (p < 0.001).

Most studies regarding risk factors for peri-implant disease have concluded that smoking is clearly involved [35-40]. This is also supported by the present study, in which a significant relationship was found between smoking and the presence of peri-implantitis. However, such data should be viewed with caution, since the group with healthy implants consisted of non-smokers; consequently, smoking could not influence the clinical, microbiological and immunological parameters studied.

Patients with peri-implantitis were significantly younger on average than patients with healthy peri-implant tissues, a finding that differs from other studies in which older patients showed higher rates of peri-implantitis [41]. In this population, when prosthetic type was studied, it was found that overdentures supported by Locator® were more frequent among patients with healthy peri-implant tissues, while overdentures on bars were more frequent on implants with peri-implantitis. In a study by Marrone et al. [41] more cases of peri-implantitis were found in patients wearing overdentures than among patients rehabilitated with fixed prostheses, which agrees with the present results. A total of 61.5% of the patients with peri-implantitis had both arches rehabilitated, compared with only 22.7% of patients with healthy peri-implant tissues, and there were no cases of implants with peri-implantitis who had undergone rehabilitation of the mandible exclusively.

P. gingivalis was detected in the half of gingivitis patients and in more than 80% of the periodontitis patients-derived samples [42]. High counts of *T. forsythia*, *P. gingivalis* and *T. denticola* have been observed in implants with peri-implantitis [9-12]. For the first time, a study demonstrated that the red complex periodontal bacterium Pg produces a concentration of Hydrogen sulfide capable of up-regulating IL-8 expression induced in gingival and oral epithelial cells, revealing a possible mechanism that may promote the inflammation in periodontal disease [43]. In the present study there was a significant relation between peri-implantitis and *P. gingivalis*, association with *P. gingivalis* and *T. denticola*, and total bacterial load. Other studies [14,44-47] found these bacteria in patients with healthy peri-implant tissue, which was similar to the present sample of healthy peri-implant patients, in which red complex was found at 11.1% of healthy peri-implant sites, while red complex was found at 25% of implants with peri-implantitis. In a study by Nowzari et al. [23] it was found that 16.7% of healthy peri-implant tissues showed presence of *P. gingivalis*, and 25% *T. forsythia*, results that are similar to this study.

One of the options for diagnosing peri-implant disease is peri-implant sulcus fluid (PISF) analysis, which offers a non-invasive means of studying the host response to peri-implant disease, and may provide an early indication of those patients at risk of developing active disease [16]. In the present study, PISF volume was greater in the healthy implants (91.7 ± 50.3) than in the peri-implantitis group (83.9 ± 43.1), though the difference was not statistically significant.

Many studies have examined the presence of cytokines in patients with periodontitis [48-50]. Due to the similarity between periodontitis and peri-implantitis, many inflammatory markers have been evaluated for monitoring peri-implant health and can indicate the presence of either disease [51-53]. Bacterial products from periodontal pathogens stimulate the production of inflammatory mediators secreted in PISF, which cause the destruction of the peri-implant tissues [17]. Certain cytokines have been proposed as potentially valid diagnostic or prognostic markers of periodontal or peri-implant tissue destruction [16]. An increase in interleukin levels is observed in patients with peri-implant disease, though there is controversy over the effect of interleukins in crevicular fluid and peri-implantitis in relation to implant failure or the development of periimplant disease [54]. The IL-10 is an anti-inflammatory cytokine, produced by T-helper 2 cells (Th2), macrophages and B cells, which inhibits synthesis of pro-inflammatory cytokines such as IL-1, IL-2, IL-6, IL-8, TNF-a and IFN-g (interferon gamma) [55]. On the other hand, IL10 acts as a B cell stimulator, enhancing B cell proliferation and differentiation [56]. These facts suggest that IL10 can play important roles in the regulation of celular and humoral immune responses [57]. As regards IL-10, Liskmann et al. (18) reported a higher concentration of IL-10 in patients with peri-implantitis. In contrast, Duarte et al. [58] observed no differences between healthy subjects and patients with disease. Some studies have previously shown interleukin-1ß (IL-1ß) in PISF to be elevated in cases of peri-implantitis [52,59,60]. TNF-α, a cytokine with some functions similar to those of IL-1β, has been detected at sites affected by periodontitis [61] TNF-α and IL-1β act synergistically to initiate the cascade of inflammatory mediators [62]. IL-6 has pro-inflammatory effects and is responsible for the collagen resorption of gingival tissues [63], while IL-10 is an inhibitor of inflammation [64]. IL-8 acts as a potent chemoattractant for neutrophils in gingival tissues [65]. In this study, it was found that IL-1β (p < 0.01), IL-6 (p < 0.01), IL-10 (p < 0.05) and TNF-α (p < 0.01) were significantly increased at the sites with peri-implantitis, while IL-8 was not.

The main component of soft and hard tissue destruction associated with periodontal disease is believed to be the result of activation of the host immunoinflammatory response to bacterial challenge [66]. IL-1 and IL-6 have both been found to be significantly elevated at diseased periodontal sites compared with healthy or inactive sites [67]. IL-1 has also been positively correlated with increased probe depth and attachment loss [68]. Other clinical data indicate that IL-6 levels are higher in refractory periodontitis, and increased granulocyte chemotactic factor (GCF) levels correlate to gram-negative fimbriae [69]. Based upon the increased expression of IL-1 and IL-6 in inflamed gingiva and high levels of GCF in periodontitis patients, several studies have suggested that an increased production of these cytokines may play an important role in periodontal tissue destruction [48].

## Conclusions

An analysis has been made of the clinical, microbiological and host response aspects in implants with peri-implantitis. The results of the present study involving a limited patient sample suggest that the peri-implant microbiota and which dental arch was rehabilitated involved could contribute to bone loss in peri-implantitis. A significant relationship is observed between the concentration of cytokines (interleukins 1β, 6 and 10 and TNF-α) and the inflammatory response in peri-implantitis tissue.

## Abbreviations

*T. forsythia*: *Tannerella forsythia*
*P. gingivalis*: *Porphyromonas gingivalis*
*T. denticola*: *Treponema denticola*
TNF-α: Tumor necrosis factor-α
IL: Interleukin
FACS: Flow cytometry
mGI: Modified gingival index
mPI: Modified plaque index
PPD: Probing pocket depth
TBL: Total bacterial load
TSA: Treatment surface avantblast
